# The Calcitriol/Vitamin D Receptor System Regulates Key Immune Signaling Pathways in Chronic Lymphocytic Leukemia

**DOI:** 10.3390/cancers13020285

**Published:** 2021-01-14

**Authors:** Marina Gerousi, Fotis Psomopoulos, Konstantia Kotta, Maria Tsagiopoulou, Niki Stavroyianni, Achilles Anagnostopoulos, Athanasios Anastasiadis, Maria Gkanidou, Ioannis Kotsianidis, Stavroula Ntoufa, Kostas Stamatopoulos

**Affiliations:** 1 Institute of Applied Biosciences, Centre for Research and Technology Hellas, 57001 Thessaloniki, Greece; mgerousi@certh.gr (M.G.); fpsom@certh.gr (F.P.); ntina_kotta@yahoo.com (K.K.); m.tsagiopoulou@certh.gr (M.T.); sntoufa@certh.gr (S.N.); 2 Medical Department, Democritus University of Thrace, 68100 Alexandroupolis, Greece; jankots@yahoo.gr; 3 Department of Molecular Medicine and Surgery, Karolinska Institute, 17177 Stockholm, Sweden; 4 Hematology Department and HCT Unit, G. Papanikolaou Hospital, 57010 Thessaloniki, Greece; stavrogianni.gpapanikolaou@n3.syzefxis.gov.gr (N.S.); achilles.gpapanikolaou@n3.syzefxis.gov.gr (A.A.); 5 Blood Transfusion Department, G. Papanikolaou Hospital, 57010 Thessaloniki, Greece; anasttha@otenet.gr (A.A.); gkanidoum@papanikolaou.gr (M.G.)

**Keywords:** calcitriol, microenvironment, vitamin D receptor, chronic lymphocytic leukemia, RNA-sequencing

## Abstract

**Simple Summary:**

Calcitriol, the biologically active form of vitamin D, modulates a plethora of cellular processes following its receptor ligation, namely the vitamin D receptor (VDR). Epidemiological studies have linked low blood levels of vitamin D to adverse disease outcome in several B cell malignancies, including chronic lymphocytic leukemia (CLL), for as yet undetermined reasons. In this study, we sought to obtain deeper biological insight into the role of vitamin D in the pathophysiology of CLL. To this end, we investigated whether the calcitriol/VDR system is functional in CLL and analyzed key signaling pathways that are regulated by calcitriol supplementation, while also exploring the role of microenvironmental signals in the regulation of calcitriol/VDR system. Overall, we provide evidence that the calcitriol/VDR system is functional in CLL, regulating signaling pathways critical for cell survival/proliferation. Although microenvironmental triggers can modulate VDR expression and function, calcitriol appears to act independently, alluding to a potential clinical utility of vitamin D supplementation in CLL.

**Abstract:**

It has been proposed that vitamin D may play a role in prevention and treatment of cancer while epidemiological studies have linked vitamin D insufficiency to adverse disease outcomes in various B cell malignancies, including chronic lymphocytic leukemia (CLL). In this study, we sought to obtain deeper biological insight into the role of vitamin D and its receptor (VDR) in the pathophysiology of CLL. To this end, we performed expression analysis of the vitamin D pathway molecules; complemented by RNA-Sequencing analysis in primary CLL cells that were treated in vitro with calcitriol, the biologically active form of vitamin D. In addition, we examined calcitriol effects ex vivo in CLL cells cultured in the presence of microenvironmental signals, namely anti-IgM/CD40L, or co-cultured with the supportive HS-5 cells; and, CLL cells from patients under ibrutinib treatment. Our study reports that the calcitriol/VDR system is functional in CLL regulating signaling pathways critical for cell survival and proliferation, including the TLR and PI3K/AKT pathways. Moreover, calcitriol action is likely independent of the microenvironmental signals in CLL, since it was not significantly affected when combined with anti-IgM/CD40L or in the context of the co-culture system. This finding was also supported by our finding of preserved calcitriol signaling capacity in CLL patients under ibrutinib treatment. Overall, our results indicate a relevant biological role for vitamin D in CLL pathophysiology and allude to the potential clinical utility of vitamin D supplementation in patients with CLL.

## 1. Introduction

Vitamin D is the precursor to the steroid hormone calcitriol, which is the biologically active form of vitamin D, and exerts its biological activities by regulating the transcriptional activation and/or repression of target genes. In order to achieve this, calcitriol binds to the vitamin D receptor (VDR), a nuclear transcription factor that is present in almost all tissues [1]. After activation by calcitriol, VDR undergoes heterodimerization with the retinoic X receptor (RXR). The activated calcitriol-VDR-RXR complex enters the nucleus and binds to vitamin D response elements (VDREs) located in the promoter regions of target genes [2]. A well-known target of VDR is CYP24A1, a 24-hydroxylase enzyme responsible for the degradation of calcitriol via a negative feedback regulation loop [3,4]. Calcitriol can also act independently of the VDR, by rapidly activating signaling molecules such as PLC, PI3K and MAP kinases [5,6].

The classical role of calcitriol concerns the physiological regulation of calcium and phosphate in the context of bone formation [7]. However, various studies have demonstrated that calcitriol also engages in multiple extra-skeletal actions, including, amongst others, the regulation of many cellular pathways that could play an important role in determining cancer risk and evolution, including the MAPK/ERK and PI3K/AKT pathways [8].

VDR is expressed by cells of the immune system, including B cells, where vitamin D was shown to inhibit the proliferation of activated B cells and induce their apoptosis [3]. Moreover, calcitriol has been found to inhibit the differentiation of B cells to memory B cells and plasma cells, leading to the inhibition of immunoglobulin (IG) production [9,10]. Interestingly, evidence exists for an association between vitamin D insufficiency and adverse outcomes in several B cell malignancies, e.g., follicular lymphoma [11], diffuse large B cell lymphoma [12], and chronic lymphocytic leukemia [13].

Chronic lymphocytic leukemia (CLL) is a malignancy of mature B cells characterized by pronounced heterogeneity regarding both the underlying biology as well as the clinical presentation and eventual outcome [14], that appear to be critically dependent on a complex interplay between microenvironmental triggering and cell-intrinsic aberrations [15]. In CLL, observational studies have reported significantly shorter time-to-treatment (TTT) and overall survival (OS) for 25(OH)D-insufficient patients [13], even amongst Binet stage A cases [16]. Interestingly, it has also been documented that vitamin D replenishment in CLL patients, including those undergoing chemotherapy, is safe and effective in restoring vitamin D levels [17,18]. Nevertheless, the above-mentioned studies concluded that thorough clinical testing will be required before reaching solid conclusions as to whether normalizing vitamin D levels may delay disease-progression of patients with early disease.

There are limited experimental data concerning the role of vitamin D and its receptor, VDR, in malignant CLL B cells. Ex vivo supplementation of a vitamin D analog (EB1089) has been found to induce apoptosis in CLL cells via a p53-independent mechanism [19]. Moreover, it has been reported that vitamin D administration in vitamin D-deficient CLL patients suppresses chemokine CCL11 levels, that confer a survival advantage on CLL cells [20]. Finally, vitamin D has also been shown to inhibit CLL cell-mediated induction of myeloid-derived suppressor cells [21].

With this background, here we sought to obtain deeper biological insight into the role of vitamin D in the pathophysiology of CLL. To this end, we investigated whether the calcitriol/VDR system is functional in CLL cells and analyzed the key molecules and signaling pathways that are regulated by calcitriol supplementation, while also exploring the role of microenvironmental signals in the regulation of calcitriol/VDR system. Our novel findings support that the calcitriol/VDR system is active in CLL and, moreover, that the effects of calcitriol appear unaffected by various microenvironmental triggers known to promote CLL cell survival and/or proliferation.

## 2. Results

### 2.1. The VDR Is Expressed and Acts as a Transcription Factor in CLL Cells

**Figure 1:**
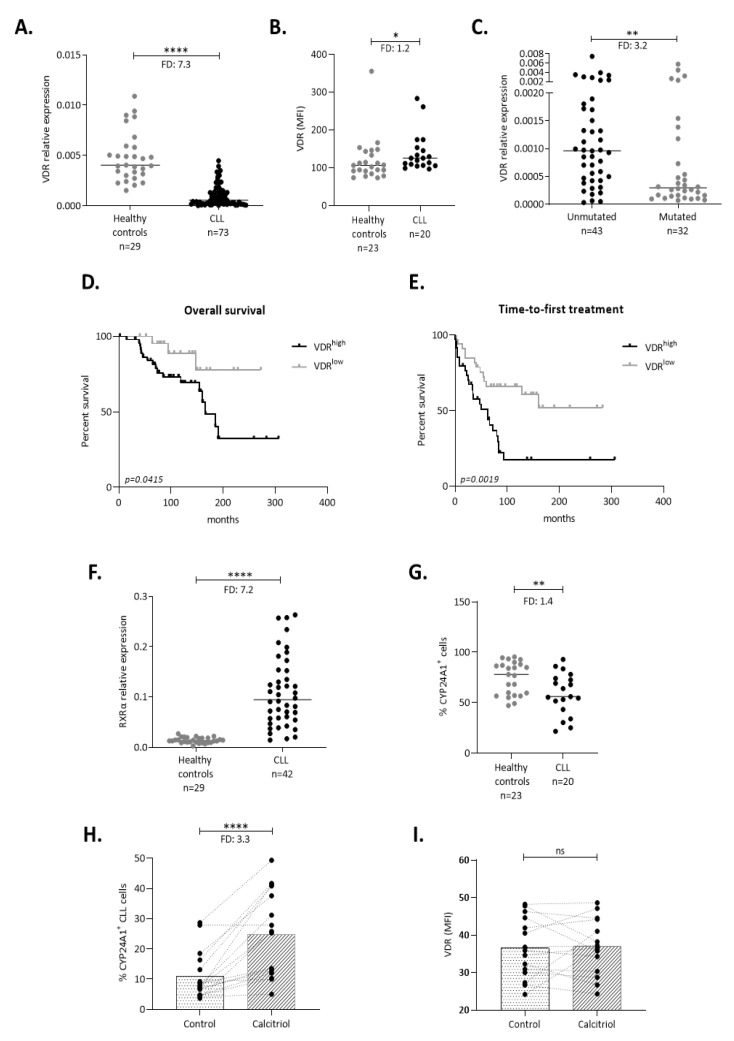
Vitamin D receptor (VDR) is underexpressed in chronic lymphocytic leukemia (CLL), albeit functional as indicated by CYP24A1 induction. (**A**) VDR mRNA expression is significantly lower in CLL compared to healthy donors’ CD19^+^ B cells. The y axis represents VDR relative expression to GAPDH as detected by RQ-PCR. (**B**) The opposite pattern is observed at the protein level, as validated by flow cytometry. The y axis represents VDR mean fluorescent intensity (MFI). (**C**) VDR mRNA expression is significantly increased in U-CLL cases in comparison with M-CLL. (**D**,**E**) Kaplan Meier OS and time-to-first treatment (TTFT) curves for CLL patients with high and low VDR mRNA expression. VDR^high^ cases show shorter overall survival (OS) (**D**) and shorter TTFT (**E**), compared to VDR^low^ cases. (**F**) RXRα is overexpressed in CLL cases in comparison with B cells from healthy donors. y axis represents RXRα relative expression to GAPDH as detected by RQ-PCR. (**G**) CLL cases show significantly lower percentage of CYP24A1^+^ cells compared to healthy cases (**A**–**C**, **F**,**G**). Each individual data point represents a unique case and lines represent the median values. The Mann–Whitney test was performed to assess statistical significance. (**H**,**I**) Calcitriol treatment for 24 h led to increased percentage of CYP24A1^+^ CLL cells (**H**) and no change in VDR expression (**I**) compared to unstimulated control cells as revealed by flow cytometric analysis. Bars represent the median values and the Wilcoxon test was performed to assess statistical significance. * *p* < 0.05, ** *p* < 0.01, **** *p* < 0.0001, FD: Fold Difference, ns: not significant.

Using qPCR analysis, we evaluated VDR gene expression in purified circulating malignant cells from 73 CLL patients as well as B cells from 29 healthy donors. VDR mRNA was found significantly underexpressed in CLL cells compared to healthy B cells (FD: 7.3; *p <* 0.0001) (Figure 1A). In contrast, this expression pattern was not confirmed at the protein level by flow cytometry (Figure 1B) disclosing marginally lower VDR levels in healthy donors versus CLL patients (FD: 1.2; *p <* 0.05), hence overall suggesting the possibility of post-transcriptional modulation of VDR expression in CLL. Interestingly, CLL cases with unmutated IGHV genes (U-CLL) showed significantly higher VDR mRNA levels compared to those with mutated IGHV genes (M-CLL) (FD: 3.2, *p <* 0.01) (Figure 1C). This association was also likely reflected in significantly shorter OS (*p* < 0.05) and time-to-first treatment (TTFT) (*p* < 0.01) for patients with high VDR mRNA levels (VDR^high^) versus those with low VDR mRNA levels (VDR^low^) (Figure 1D,E) (Table S1).

We then assessed the mRNA expression of *RXRα*, the VDR co-receptor, and found significantly increased levels in CLL compared to healthy B cells (FD: 7.2, *p* < 0.0001) (Figure 1F), albeit with no differences between U-CLL and M-CLL cases (Figure S1A). Finally, we evaluated by flow cytometry the protein expression of *CYP24A1*, a well-known target gene of the activated VDR, and found significantly lower percentages of CYP24A1^+^ cells in CLL cases versus healthy donors (FD: 1.4, *p* < 0.01) (Figure 1G).

Next, we sought to determine if the VDR receptor is functional after binding its ligand. To this purpose, we cultured CLL cells with calcitriol for 24 h and measured by flow cytometry the expression of *CYP24A1.* We found that the percentage of CYP24A1^+^ CLL cells dramatically increased in the presence of calcitriol compared to non-treated cells (n = 16 cases: FD: 3.3, *p* < 0.0001; Figure 1H), indicating that the VDR is functional in CLL. U-CLL cases (*n* = 9) displayed stronger induction of CYP24A1 (FD: 4, *p <* 0.01) after calcitriol treatment compared to M-CLL cases (*n* = 7) (FD: 1.9, *p* < 0.05) (Figure S1B,C). Interestingly, VDR expression remained unchanged after in vitro calcitriol supplementation, as revealed by flow cytometric analysis (*n* = 15 cases) (Figure 1I); this was consistent in both U-CLL and M-CLL cases (Figure S1D,E). Of note, CLL cell viability was not altered by calcitriol treatment (Figure S2). Overall, these results indicate that, despite lower expression compared to normal B cells, the VDR is functional in CLL cells, as evidenced by calcitriol-mediated CYP24A1 induction.

### 2.2. Calcitriol Induces a Distinctive Transcriptional Program in CLL Cells

**Figure 2:**
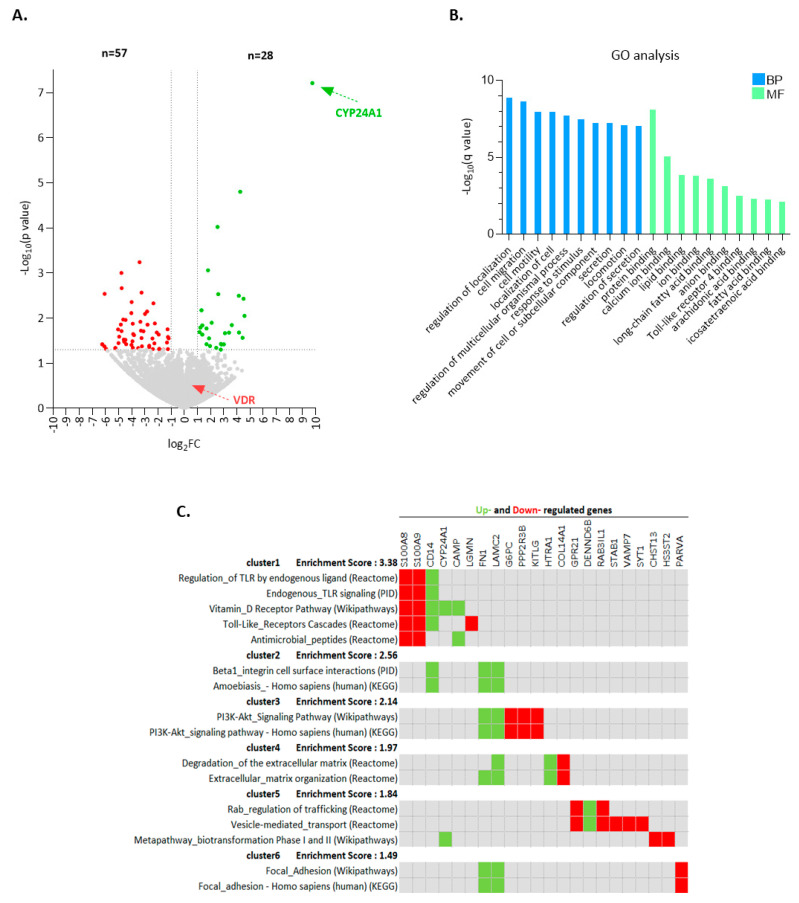
Calcitriol-treated CLL cells display a distinct transcriptional profile. (**A**) Volcano plot indicating the differentially expressed genes in calcitriol-treated vs untreated CLL cells. The fold change of gene expression is displayed on the x-axis and the significance of difference in gene expression [(-log10(*p* value)] is shown on the y-axis. The significantly upregulated genes are highlighted in green, whereas, the significantly downregulated genes are highlighted in red. (**B**) GO enrichment analysis showing top 10 significantly enriched biological processes (BP) and molecular functions (MF) in calcitriol-treated CLL cells. (**C**) The figure presents enriched pathways from the pathway enrichment analysis (*q* value < 0.1) and the differentially expressed genes between calcitriol-treated and non-treated CLL cases that are implicated. Upregulated genes are illustrated in green, whereas downregulated genes are shown in red. Enrichment score for each cluster is considered the overall enrichment score for the group based on the *p* value of each term members.

RNA-Sequencing (RNA-Seq) was performed in six CLL cases after ex vivo treatment with calcitriol for 24 h. Differential expression analysis revealed 85 differentially expressed genes (DEGs) (log2FC ≥ |1| and *p ≤* 0.05), of which 28 (32.9%) were overexpressed in calcitriol-treated cells versus unstimulated CLL cells, thus, contrasting the remaining 57 (67.1%) which showed the opposite pattern (Figure 2A, Table S2). RNA-Seq analysis confirmed our aforementioned findings that the *CYP24A1* gene is significantly upregulated (log2FC = 9.7, *p* = 6.12 × 10^−8^) by calcitriol, being the top upregulated gene, whereas the *VDR* gene remained unaffected by this treatment (Figure 2A).

Supervised hierarchical clustering analysis was performed, based on the differentially expressed genes, revealing distinct gene expression patterns between calcitriol-treated versus untreated (control) CLL cells (Figure S3). Gene ontology (GO) enrichment analysis unveiled pronounced enrichment (*q* value *<* 0.1) in biological processes related to the regulation of localization, cell migration and motility, locomotion, and secretion. Concerning the molecular functions, the most enriched terms related to binding, including protein binding, calcium ion binding, lipid binding, and Toll-like receptor (TLR) 4 binding (Figure 2B).

Moreover, pathway enrichment analysis revealed that, besides the VDR pathway, calcitriol-regulated genes are implicated in TLR cascades and the PI3K/AKT signaling pathway, both known to be deregulated in CLL biology [22,23]. Finally, degradation of the extracellular matrix and vesicle-mediated transport were also among the calcitriol-regulated pathways (Figure 2C). Interestingly, genes known to be implicated in lymphoma biology, such as *S100A8*, *S100A9* and *KITLG* [24,25], were included amongst those regulated by calcitriol.

Altogether, transcriptome analysis supported VDR functionality in CLL and highlighted the possible implication of calcitriol in the regulation of immune signaling pathways relevant to CLL pathophysiology.

### 2.3. Calcitriol Affects Key Immune Signaling Cascades in CLL Cells

**Figure 3:**
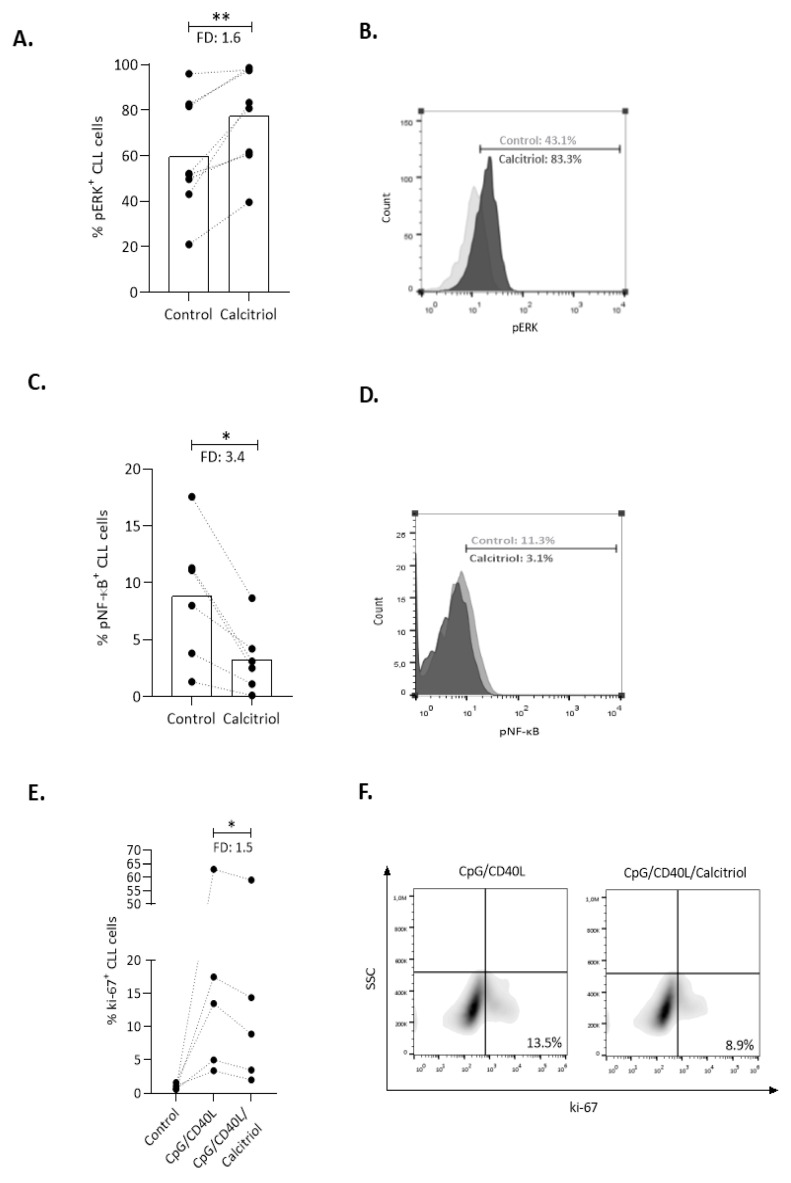
Calcitriol modulates key signaling molecules and inhibits CLL cell proliferation. (**A**) Calcitriol treatment for 24 h led to increased percentage of pERK^+^ CLL cells. (**B**) Representative histograms from flow cytometry analysis for pERK^+^ cells in control and calcitriol-treated CLL cells for one CLL case. (**C**) Decreased percentage of pNF-κB^+^ CLL cells compared to unstimulated control cells after calcitriol supplementation; (**D**) representative case. (**E**) Flow cytometric analysis of proliferating cells (Ki-67^+^) in U-CLL cases treated with CpG/CD40L for four days supplemented with calcitriol at 48 h of culture. (**F**) Representative dot plot showing the decrease in Ki-67^+^ cells after calcitriol supplementation. Bars represent the median values and the Wilcoxon test was performed to assess statistical significance. * *p* < 0.05, ** *p* < 0.01, FD: Fold Difference.

We further investigated the impact of calcitriol on signaling pathways known to regulate CLL cell proliferation and survival. Considering that calcitriol regulates the TLR and PI3K/AKT signaling pathways, we sought to investigate the activation status of extracellular signal-regulated kinase 1/2 (ERK1/2) and NF-κB p65, both implicated in these pathways. To this end, using intracellular phospho-flow cytometry, we analyzed pERK1/2 and pNF-κΒ p65 levels in CLL cells supplemented with calcitriol for 24 h and observed a significant increase in pERK (*n* = 8, FD: 1.6, *p* < 0.01) (Figure 3A,B), hence contrasting the phosphorylation of NF-κB that was significantly decreased (*n* = 6, FD: 3.4, *p < 0.05*) (Figure 3C,D).

Next, we investigated the impact of calcitriol on the functionality of the TLR pathway by performing concurrent stimulation of CD19^+^ B cells from U-CLL cases via the TLR9 and CD40 receptors in order to induce CLL cell proliferation. On day 2, calcitriol was added to the culture medium and, on day 4, the percentage of proliferating (Ki-67^+^) CLL cells was determined by flow cytometry. CpG/CD40L stimulation induced CLL cell proliferation as expected [26]. CpG/CD40L-stimulated cells supplemented with calcitriol showed significantly reduced proliferation compared to cells treated with CpG/CD40L alone (*n* = 5, FD: 1.5, *p < 0.05*) (Figure 3E,F). Altogether, these results support the notion that calcitriol can regulate key signaling pathways implicated in CLL pathophysiology.

### 2.4. Mincroenvironmental Signals Affect VDR Signaling, yet Do not Impede the Action of Calcitriol in CLL

**Figure 4:**
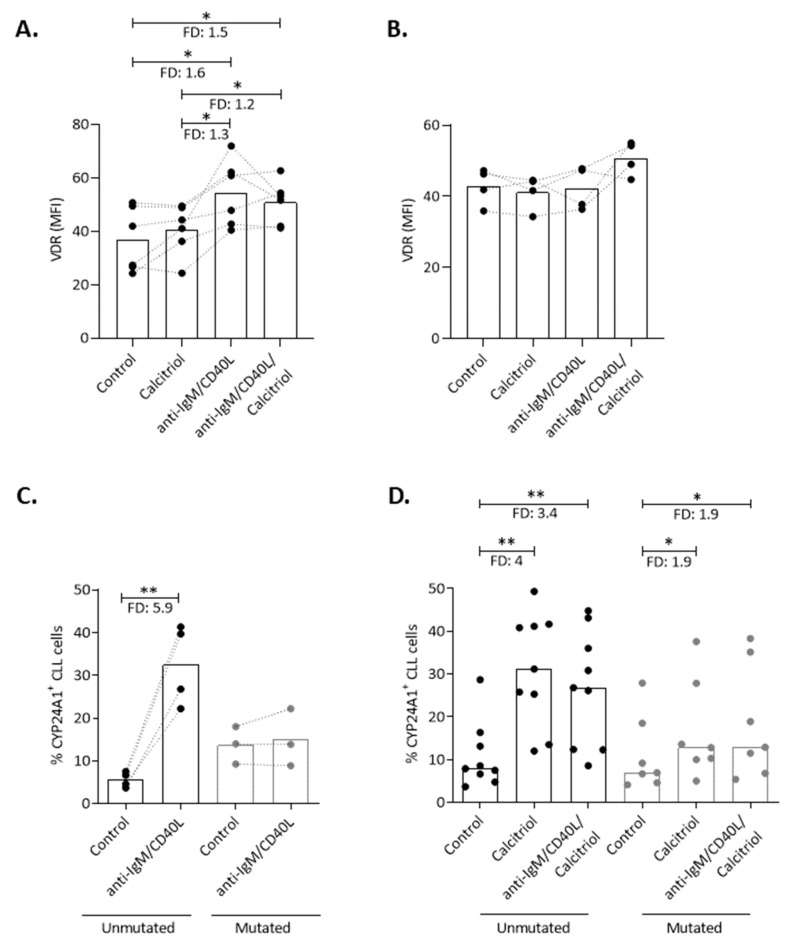
Immune signals affect VDR/calcitriol system, yet calcitriol action remains unaffected. Flow cytometric analysis of VDR (MFI) in (**A**) CLL with unmutated IGHV genes (U-CLL) and (**B**) CLL with mutated IGHV genes (M-CLL) cases treated with calcitriol, anti-IgM/CD40L, and a combination of them for 24 h. (**C**) Flow cytometric analysis of CYP24A1 expression in U-CLL and M-CLL cells treated with anti-IgM/CD40L for 24 h. (**D**) U-CLL and M-CLL cases were incubated with calcitriol and combination of anti-IgM/CD40L/calcitriol for 24 h. The percentage of CYP24A1^+^ CLL cells was determined by flow cytometry. (**A**–**D**) Bars represent the median values and the Wilcoxon test was performed to assess statistical significance. * *p* < 0.05, ** *p* < 0.01, FD: Fold Difference.

In order to investigate the impact of external triggering on VDR and CYP24A1 expression, we performed concurrent stimulation of CD19^+^ B cells from U-CLL and M-CLL cases via the BcR and CD40 receptors for 24 h. Flow cytometric analysis showed that the VDR was significantly induced by co-stimulation with anti-IgM/CD40 ligand in U-CLL (*n* = 6, FD: 1.6, *p <* 0.05) (Figure 4A), while no changes were observed in M-CLL (*n* = 4; Figure 4B). The combination of calcitriol with anti-IgM/CD40L induced changes similar to BcR/CD40 stimulation alone in both U-CLL and M-CLL (Figure 4A,B). BcR/CD40 stimulation led to a significant increase in CYP24A1^+^ cells in U-CLL (*n* = 4, FD: 5.9, *p* < 0.01) but not in M-CLL (*n* = 3) (Figure 4C), yet co-stimulation of CLL cells with calcitriol/anti-IgM/CD40 ligand did not confer any additional change compared to calcitriol alone both in U-CLL and M-CLL (Figure 4D). Overall, BcR/CD40 stimulation appears to modulate the VDR signaling pathway exclusively in U-CLL upregulating the expression levels both the VDR and CYP24A1, though calcitriol seems to act independently of these microenvironmental triggers since its action was not impeded when combined with anti-IgM/CD40L stimulants.

**Figure 5:**
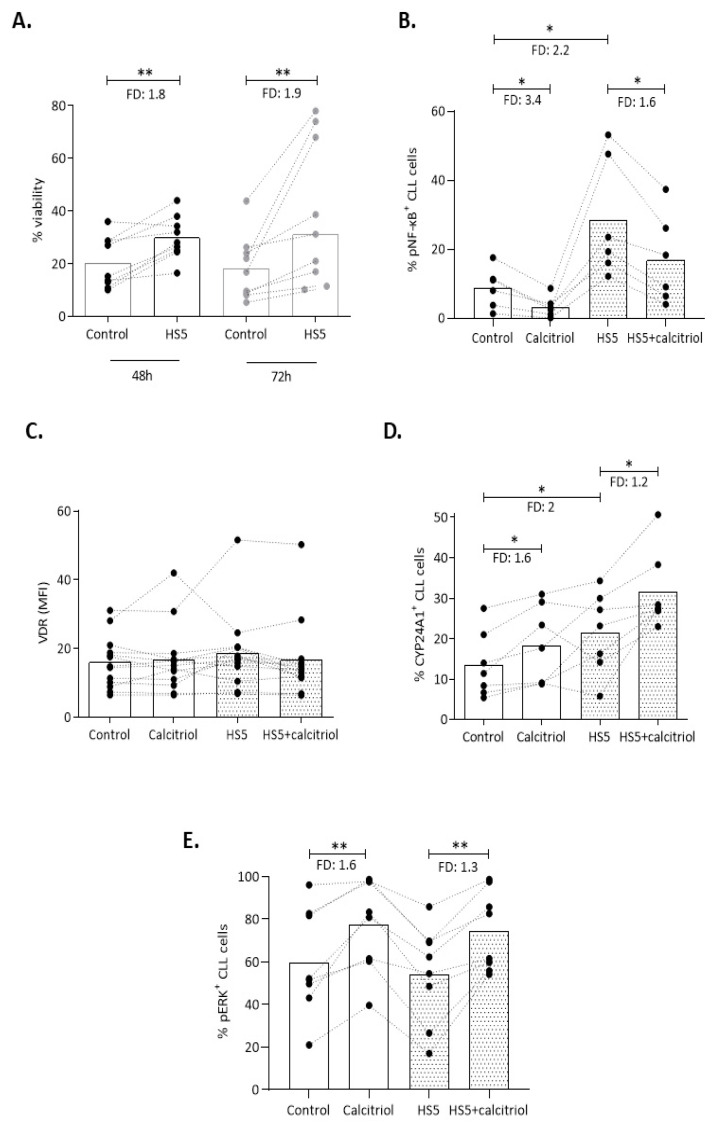
Co-culture of CLL cells with HS5 mesenchymal cells affects VDR signaling pathway, but not calcitriol action. (**A**) Viability of mono- vs co-cultured CLL cells was assessed by flow cytometry at 48 and 72 h of cell culture. (**B**–**E**) CLL cells from U-CLL cases were co-cultured with the HS5 stromal cell line and both mono- and co-cultured CLL cells were treated with calcitriol for 24 h. (**B**) pNF-κB^+^ cells, (**C**) VDR (MFI), (**D**) CYP24A1^+^, and (**E**) pERK^+^ cells were determined by flow cytometry. Bars represent the median values and the Wilcoxon test was performed to assess statistical significance. * *p* < 0.05, ** *p* <0.01, FD: Fold Difference.

Next, we co-cultured CLL cells with the HS5 bone marrow stromal cell line, that partially mimics the microenvironment providing stimuli that prevent apoptosis of CLL cells ex vivo [27]. Considering the herein documented limited responsiveness of M-CLL cells to calcitriol, only U-CLL cases were selected for the co-culture system. Co-cultured CLL cells from nine U-CLL cases showed significantly higher viability in comparison to mono-cultured CLL cells for 48 and 72 h (FD: 1.8; FD: 1.9, *p <* 0.01 respectively) (Figure 5A) and significantly increased pNF-κB levels (FD: 2.2, *p <* 0.05) (Figure 5B), as previously described [28]. VDR expression was not altered in the co-culture system (*n* = 13), even when calcitriol was applied (Figure 5C). On the contrary, the presence of stromal cells in the culture system resulted in a significant increase in CYP24A1^+^ CLL cells at 24 h (*n* = 9, FD: 2, *p <* 0.05), which was further enhanced when calcitriol was added to the medium (FD: 1.2, *p <* 0.05) (Figure 5D), indicating that calcitriol action is maintained in the co-culture system. Supporting this argument, the addition of calcitriol to the co-culture system induced pERK levels (*n* = 8, FD: 1.3, *p <* 0.01) (Figure 5E) and strongly reduced HS5-induced NF-κB p65 phosphorylation (*n* = 6, FD: 1.6, *p* < 0.05) (Figure 5B) as in mono-cultured CLL cells. Moreover, using our co-culture system, we performed a proliferation assay for two U-CLL cases in the presence of calcitriol. On day 2, calcitriol was added to the culture medium and, on day 4, the percentage of proliferating (Ki-67^+^) CLL cells was determined by flow cytometry. CLL co-cultured cells treated with calcitriol showed reduced proliferation compared to untreated cells (Figure S4).

### 2.5. Preserved Calcitriol/VDR Signaling Capacity in CLL Cases under Ibrutinib Therapy

**Figure 6:**
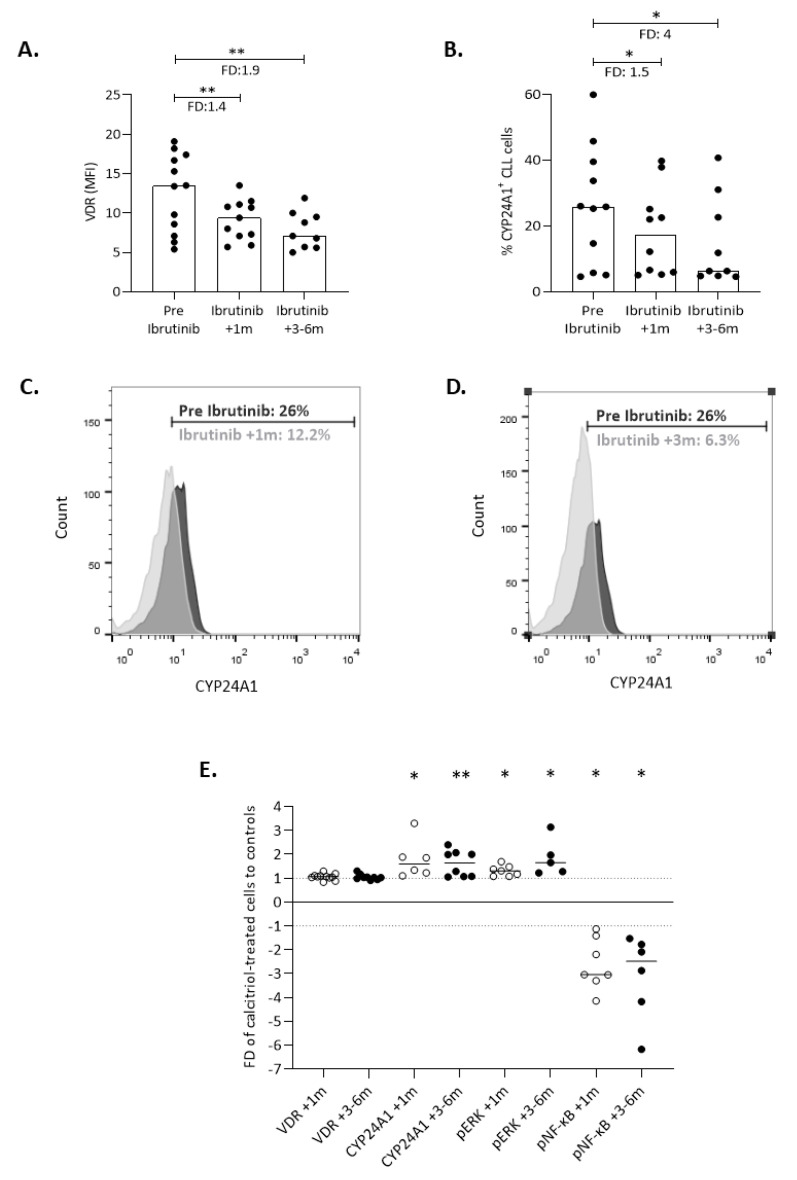
The calcitriol/VDR system appears unaffected by inhibition (ibrutinib) of microenvironmental signals. (**A**,**B**) CLL cells from patients before treatment initiation with ibrutinib and at +1m and +3–6 m under treatment were evaluated for VDR and CYP24A1 expression by flow cytometry. Bars represent the median values and the Wilcoxon test was performed to assess statistical significance. (**C**,**D**) Representative histograms from flow cytometry analysis for CYP24A1^+^ cells (**C**) at pre-treatment versus at +1 m of ibrutinib treatment and (D) at pre-treatment versus at +3–6 m of ibrutinib treatment. (**E**) VDR (MFI), CYP24A1 (% +), pERK (% +) and pNF-κB (% +) of CLL cells treated with calcitriol. Individual data points represent the fold difference (FD) of calcitriol-treated cells to the untreated control cells and the horizontal lines show the median value. Asterisks above each time point represent statistically significant differences compared to the untreated controls. * *p* < 0.05, ** *p* < 0.01.

Next, we investigated whether the BTK inhibitor ibrutinib, an agent interfering with microenvironmental interactions in CLL [29], might modulate the function of the calcitriol/VDR system by assessing VDR and CYP24A1 expression in 12 CLL patients under ibrutinib treatment. Three consecutive time points were selected; before treatment initiation, at 1 month (+1 m), and at 3–6 months (+3–6 m) under treatment. Flow cytometric analysis revealed that ibrutinib treatment led to a significant decrease in VDR levels both at +1 m (FD: 1.4, *p <* 0.01) and at +3–6 m (FD: 1.9, *p <* 0.01) in comparison to the respective pre-treatment samples (Figure 6A). Consistent with this, the percentage of CYP24A1^+^ cells was also reduced at +1 m (FD: 1.5, *p<* 0.05), with further reduction noted at +3–6 m (FD: 4, *p <* 0.05) (Figure 6B,D).

Moreover, CLL cells from patients under ibrutinib treatment, at both +1m and +3–6 m, were ex vivo exposed to calcitriol for 24 h. VDR levels were not affected by the addition of calcitriol at either timepoint. On the contrary, calcitriol treatment led to significantly increased CYP24A1 levels at +1m (FD: 1.6, *p <* 0.05) and +3–6 m (FD: 1.6, *p <* 0.01). Phosphorylated ERK was also increased after calcitriol administration at both timepoints (FD: 1.3; FD: 1.6, *p <* 0.05 respectively), while the phosphorylation of NF-κB was reduced at both +1 (FD: 3, *p <* 0.05) and +3–6 m (FD: 2.5, *p <* 0.05) (Figure 6E). Altogether, these findings indicate that inhibition of immune signaling by ibrutinib does not affect calcitriol action in CLL.

## 3. Discussion

In the present study, we addressed the question whether the calcitriol/VDR system is functional in CLL cells, investigated the biological processes and signaling pathways that might be regulated by calcitriol supplementation, and also explored the role of microenvironmental signals in the regulation of the calcitriol/VDR system.

VDR mRNA expression in CLL cells was lower compared to B cells from healthy donors. That said, U-CLL displayed stronger VDR expression compared to M-CLL and, most importantly, higher VDR expression was found to be significantly associated with reduced overall survival and time-to-first treatment. On the contrary, RXRα, the VDR co-receptor, was expressed at similar levels in U-CLL and M-CLL cases and overexpressed in comparison to healthy B cells, in line with the literature [30].

Stimulation of CLL B cells with calcitriol induced *CYP24A1*, a target gene of activated VDR, indicating that VDR has a functional classical action as a transcription factor in CLL cells. CYP24A1 induction was greater in U-CLL versus M-CLL cases, perhaps linked to the increased VDR levels in the former. However, VDR expression was not affected by calcitriol supplementation in either U-CLL nor M-CLL, in contrast with normal B cells, where published data show that calcitriol administration leads to increased VDR expression [3]. Additionally, calcitriol supplementation in CLL cells induced ERK phosphorylation whereas it downregulated NF-κB p65 phosphorylation, indicating that the classical action of calcitriol to modulate gene transcription might also be complemented by its ability to regulate cytoplasmic signaling cascades. Non genomic actions of calcitriol are hypothesized to involve binding to cytosolic VDR and membrane VDR, and speculated to activate the mitogen-activated ERK1/2 cascade [8]. Calcitriol-induced ERK phosphorylation has been previously reported in promyelocytic NB4 leukemia cells and T helper 17 cells [31,32], while in CLL an opposite effect has been observed, though a different vitamin D analog was used in that study (EB1089) [19]. Vitamin D-regulated NF-κB inhibition has also been reported in macrophages, suppressing TLR-mediated inflammation [33].

Delving into the mechanisms of calcitriol/VDR modes of action, our transcriptome analysis revealed that several genes regulated by calcitriol administration are participating in biological processes related to cell migration and motility, both mandatory for cancer progression [34]. Interestingly, these processes have been reported to be inhibited by vitamin D in breast, ovarian, and colon cancer [35,36,37]. Transcriptome analysis also revealed TLR and PI3K/AKT signaling regulation by calcitriol; this is in line with our finding that calcitriol regulates MAPK and NF-κB cascades, since ERK is activated by TLR signaling [38], while pNF-κΒ is implicated in both PI3K/AKT and TLR signaling cascades [38,39], and both regulate important cellular processes concerning cancer cell proliferation and inflammation [40,41]. Supporting this result, we here show that calcitriol can inhibit TLR/CD40-induced CLL cell proliferation. Furthermore, calcitriol-induced downregulation of *S100A8* and *S100A9* genes, that are known to promote inflammatory processes [42], as well as upregulation of *CD14*, that recognizes pathogen-associated molecular patterns [43], implies that calcitriol mediates anti-inflammatory responses in CLL. Relevant to mention, *S100A9* is overexpressed in CLL, increases with disease progression, and correlates with NF-κB pathway activation [24]. Our findings are also in accordance with published evidence for calcitriol-mediated inhibition of inflammation via impairment of TLR signaling [44]. Moreover, calcitriol has been shown to inhibit PI3K/AKT/mTOR signaling in a diffuse large B cell lymphoma (DLBCL) cell line and the HL60 cell line [45]. Considering the above and the downregulation of the NF-κB p65 phosphorylation, as a result of calcitriol supplementation, that we show here, it is not unreasonable to argue that calcitriol could act as a potential PI3K/AKT inhibitor in CLL.

CLL cells are highly dependent on the tumor microenvironment for their survival and proliferation. Our work suggests that microenvironmental triggers affect also the calcitriol/VDR system, since the prosurvival stimulants anti-IgM/CD40L modulate both CYP24A1 and VDR levels. This effect was observed exclusively in U-CLL, perhaps related to our finding of higher VDR expression in U-CLL versus M-CLL. Moreover, co-culturing CLL cells with the HS5 stromal cell line also increased CYP24A1 levels, altogether indicating that various stimulants, besides calcitriol, may regulate CYP24A1 expression. In any case, our present findings suggest that calcitriol action is likely independent of the microenvironmental signals in CLL, since it was not impeded when combined with anti-IgM/CD40L or in the context of the co-culture system.

The regulation of calcitriol/VDR system by external triggering in CLL is further supported by our finding that CLL patients under ibrutinib treatment show reduced VDR and CYP24A1 expression. Importantly, calcitriol/VDR signaling capacity was maintained in CLL cells under ibrutinib since calcitriol administration to ibrutinib-treated cells could upregulate CYP24A1 and pERK and decrease pNF-κB, further supporting our argument that microenvironmental signaling does not impede calcitriol action.

From a clinical perspective, the findings reported herein also raise the intriguing hypothesis that calcitriol supplementation might have a place in CLL patients under ibrutinib and/or other treatment modalities. Support to this hypothesis is provided by (i) associations between pretreatment vitamin D deficiency and inferior progression-free and overall survival in Hodgkin lymphoma [46]; and, (ii) preclinical evidence in non-Hodgkin lymphoma that vitamin D replenishment can lead to improved rituximab-mediated cellular cytotoxicity in vitro [12]. In CLL, a clinical trial carried out by the Mayo Clinic showed that vitamin D substitution in CLL patients with insufficient vitamin D levels is safe and feasible, however could not provide evidence that vitamin D can delay disease progression [17]. More recently, the combination of vitamin D with curcumin was reported to be safe in previously untreated patients with early stage CLL or small lymphocytic lymphoma (SLL), yet event-free and overall survival did not show any alteration [47]. Obviously, therefore, further studies are required in order to reach definitive conclusions regarding the role, if any, of vitamin D in the management of patients with B cell malignancies, including CLL.

## 4. Materials and Methods

### 4.1. Study Group

Peripheral blood samples from 94 patients diagnosed with CLL, according to the guidelines of the International Workshop Chronic Lymphocytic Leukemia/National Cancer Institute (iwCLL/NCI) [48], were obtained with patients’ informed consent. Patients were either treatment-naïve or off treatment at least 6 months prior to sample collection, excepting the ibrutinib study group, which was analyzed at 1 month and 3–6 months after treatment initiation. Clinicobiological data for the study group are listed in Table S3. Moreover, peripheral blood samples were collected after informed consent from 29 age-matched healthy donors. The study was approved by the Ethics Committee of CERTH (decisions: ETH.COM-1| 2 July 2014; and ETH.COM-45 | 21 March 2019) and conducted in accordance with the Declaration of Helsinki.

### 4.2. Cell Isolation and Cell Cultures

CD19^+^ cells were negatively selected from whole blood from CLL patients and healthy donors using the RosetteSep B-cell enrichment kit (StemCell Technologies, Vancouver, Canada) according to the manufacturer’s instructions. The purity of the samples was assessed by flow cytometry and the percentage of CD19^+^ cells always exceeded 95%.

Both CLL cells and the HS5 stromal cell line (ATCC^®^ CRL-11882™) were cultured in RPMI 1640 medium (Sigma-Aldrich, St. Louis, MO, USA) supplemented with L-glutamine, 10% heat-inactivated fetal bovine serum (FBS), 50 μg/mL penicillin/streptomycin and 15 μg/mL gentamicin. All cultures were incubated at 37°C in a humidified 5% CO_2_ atmosphere. The HS5 cell line was a kind gift from Prof. Asterios S Tsiftsoglou (Aristotle University of Thessaloniki, Thessaloniki, Greece).

CLL cells were cultured at a density of 3 × 10^6^ cells/mL in the presence of different stimulants for certain time points, as detailed below: (i) BcR stimulation: 10 μg/mL goat F(ab)2 anti-human IgM (ThermoFisher Scientific, Waltham, MA, USA); (ii) CD40 stimulation: soluble 0.1 μg/mL CD40L plus 1 μg/mL enhancer (Enzo Life Sciences); (iii) TLR9 stimulation: 2.5 μg/mL CpG ODN 2006 (Invivogen, San Diego, CA, USA); and (iv) VDR stimulation: 100 nM calcitriol (Selleckchem, Houston, TX, USA).

Regarding the co-culture system, 5 × 10^4^ HS5 cells/mL were seeded per well in a 24-well plate. Three days later, the confluence of the HS5 cell layer was checked and CLL cells were added at a ratio of 60:1 (3 × 10^6^ cells/mL). CLL cells were treated with DMSO (vehicle control) or calcitriol (100 nM). Cells were harvested at the indicated time points with mild trypsinization (ThermoFisher Scientific).

### 4.3. Quantification of VDR and RXRα mRNA Expression

Total cellular RNA was isolated from purified CLL B cells and healthy donors’ B cells with the NucleoSpin RNA Kit (Macherey-Nagel, Düren, Nordrhein-Westfalen, Germany), including a DNAase digestion step. Quantification of *VDR* and *RXRα* mRNA levels were achieved by RQ-PCR.

One microgram of total RNA was reverse transcribed to cDNA and a 2.5:50 aliquot of the RT product was used as the template for RQ-PCR. Quantification of *VDR* and *RXRα* mRNA levels were achieved by RQ-PCR, using custom designed primers and EvaGreen qPCR mix (Solis Biodyne, Tartu, Estonia). The *GAPDH* gene was used as reference (housekeeping gene). Gene primer sequences used were VDR: 5′-CCTTCTGTGACCCTAGAGCTGTCC-3′ and 5′-TCATCTTAGCAAAGCCAATGACCT-3′; RXRα: 5′-TTCGCTAAGCTCTTGCTCCG-3′ and 5′-AGGTGTCAATGGGTGTGTCC-3′; GAPDH: 5′-ACTGTGGATGGCCCCTCCGG-3′ and 5′-ACGGCAGGTCAGGTCCACCA-3′. For RQ-PCR experiments all samples were run in triplicate. Data were analyzed using the 2^−ΔΔCt^ method [49].

### 4.4. RNA Sequencing

Total cellular RNA was extracted from calcitriol-treated and non-treated CLL cells from 6 patients. RNA was extracted using NucleoSpin RNA kit (Macherey-Nagel) according to the manufacturer’s instructions. One μg of total RNA was used for poly(A) mRNA selection using NEBNext Poly(A) mRNA Magnetic Isolation Module. Library preparation was performed with the NEBNext Ultra II Directional RNA Library Prep Kit. The libraries were paired-end sequenced on the NextSeq 500 Illumina platform using the NextSeq 550 System Mid-Output Kit (Illumina, San Diego, CA, USA). Data analysis was performed based on the ‘new tuxedo’ protocol [50].

Reads were trimmed using cutadapt (version 1.8.1) and then mapped to the hg19 UCSC reference genome using HISAT2 (version 2.0.5). The alignments were then passed to StringTie, which assembles and quantifies the transcripts in each sample. Additionally, read quantification at the gene level was performed using htseq-count (version 0.6.0) as well as featureCounts (subread suite, version 1.6.4).

Differential expression between calcitriol-treated and non-treated CLL cells was assessed using DESeq2 (Bioconductor, 10.18129/B9.bioc.DESeq2). Genes with log2 fold change (log2FC) ≥ |1| and *p ≤* 0.05 were considered as significantly differentially expressed. Hierarchical clustering was applied to assess distinct expression profiles based on the values of all significantly differentially expressed genes. “Heatmapper” software was used for that purpose [51]. Gene ontology and pathway enrichment analyses were performed using the online tools “GenCLiP3” [52] and “gProfiler” [53]. RNA sequencing datasets have been deposited in the GEO database (accession number: GSE162427).

### 4.5. Flow Cytometry Studies 

Cell viability, proliferation, apoptosis, intracellular expression of VDR and CYP24A1 as well as the expression of phospho-ERK (pERK) and phospho-NF-κB p65 (pNF-κB) were determined by flow cytometry. In all experiments, the BD Horizon Fixable viability stain 660 (BD Biosciences, Franklin Lakes, NJ, USA) was used for gating viable cells. Co-cultured CLL cells were further stained for surface CD19 (PE-Cy5 mouse anti-human CD19 antibody, BD Biosciences) in order to be gated against the HS5 cells. Intracellular staining of VDR and CYP24A1 was performed with the Fixation/Permeabilization Solution Kit (BD Biosciences) following the manufacturer’s instructions. Intracellular staining of pERK and pNF-κB was carried out following the phosflow protocol from BD Biosciences. The list of the antibodies used is given in Table S4. Apoptosis was measured using the FITC Annexin V Apoptosis Detection Kit with PI (Immunostep, Salamanca, Spain), whereas cell proliferation was measured using FITC Mouse Anti-Ki-67 Set (BD Biosciences).

Flow cytometric analysis was conducted on a BD FACSCalibur flow cytometer (BD Bioscience). For each sample, 10,000 events were acquired and nonspecific binding was excluded by using appropriate isotypic negative control antibodies. Gating analysis strategy included only viable cells and was performed using FlowJo software (BD Biosciences).

### 4.6. Statistical Analysis

The statistical significance of differences between groups of variables and matched samples was assessed with the use of Mann-Whitney test and the Wilcoxon test, respectively. Descriptive statistics for quantitative variables included statistical measures like mean, median, and SD. Values less than 0.05 were considered statistically significant.

Survival analysis was conducted to assess the impact of VDR on overall survival (OS) and TTFT. In particular, the “survminer” R package was used to determine the optimal cutpoint for *VDR* mRNA levels, leading to the generation of two groups of CLL patients: one with high *VDR* mRNA levels (VDR^high^) and one with low *VDR* mRNA levels (VDR^low^). To compare the OS and TTFT in both groups (high vs. low), Kaplan-Meier curves were plotted and p values were calculated. All statistical analyses were performed with the use of the GraphPad Prism 8 software (La Jolla, CA, USA), and R.

## 5. Conclusions

Our study shows that the calcitriol/VDR system is functional in CLL cells, regulating signaling pathways critical for cell survival and proliferation. Although external triggers can modulate VDR expression and function in CLL, calcitriol acts independently and remains unaffected by microenvironmental signaling, prompting further investigations into the potential clinical utility of vitamin D supplementation in CLL patients under treatment.

## Data Availability

The data presented in this study are openly available in the GEO database (accession number: GSE162427) and can be found here: http://www.ncbi.nlm.nih.gov/geo/query/acc.cgi?acc=GSE162427.

## Supplementary Materials

The following are available online at https://www.mdpi.com/2072-6694/13/2/285/s1, Figure S1: RXRα expression based on somatic hypermutation status. CYP24A1 and VDR expression, after calcitriol administration, based on somatic hypermutation status, Figure S2: CLL cell viability after calcitriol treatment, Figure S3: Transcriptome analysis, Figure S4: Proliferation assay, Table S1: Survival analysis for the correlation of VDR mRNA expression with Overall Survival (OS) and Time-To-First Treatment (TTFT) in CLL cases, Table S2: List of all 85 significantly up- and down-regulated genes in CLL cells following treatment with calcitriol, Table S3: Clinicobiological data for the CLL patients’ study group, Table S4: List of antibodies used for flow cytometry analysis.
