# Supplementary material for: The Calcitriol/Vitamin D Receptor System Regulates Key Immune Signaling Pathways in Chronic Lymphocytic Leukemia
Source: Cancers (Basel). 2021 Jan 14;13(2):285. doi: 10.3390/cancers13020285 (PMC7828837; doi:10.3390/cancers13020285)
Supplement: Supplementary file 1 [file cancers-13-00285-s001.pdf]

Supplementary Materials: The Calcitriol/Vitamin D Receptor System Regulates Key Immune Signaling Pathways in Chronic Lymphocytic Leukemia

Marina Gerousi, Fotis Psomopoulos, Konstantia Kotta, Maria Tsagiopoulou, Niki Stavroyianni, Achilles Anagnostopoulos, Athanasios Anastasiadis, Maria Gkanidou, Ioannis Kotsianidis, Stavroula Ntoufa and Kostas Stamatopoulos

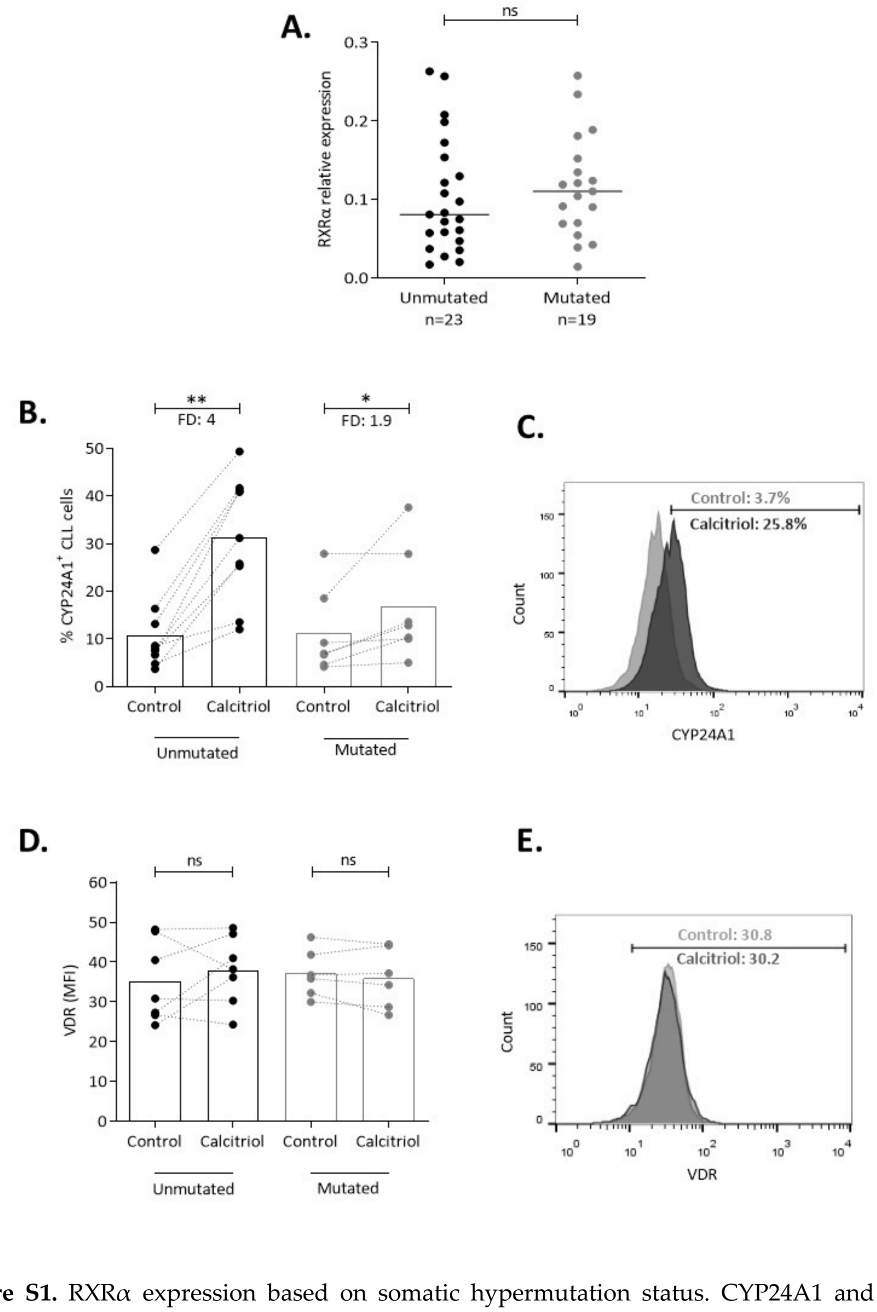

Figure S1. RXRα expression based on somatic hypermutation status. CYP24A1 and VDR expression, after calcitriol administration, based on somatic hypermutation status (**A**) No difference was recorded between U-CLL and M-CLL cases concerning RXRα mRNA expression. Lines represent the median values and the Mann-Whitney test was performed to

assess statistical significance. (**B**) The percentage of CYP24A1⁺ CLL cells was found to augment after calcitriol administration for 24 h, in both U-CLL and M-CLL cases, yet the induction is higher in U-CLL. (**C**) Representative histograms from flow cytometry analysis for CYP24A1⁺ cells in control and calcitriol-treated CLL cells for one U-CLL case. (**D, E**) VDR MFI does not change with calcitriol supplementation; (**E**) representative case. (**B, D**) Bars represent the median values and the Wilcoxon test was performed to assess statistical significance. * $p < 0.05$, ** $p < 0.01$, FD: Fold Difference, ns: not significant.

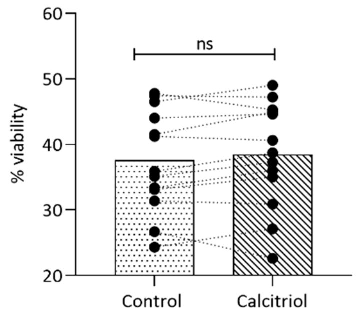

Figure S2. CLL cell viability after calcitriol treatment**.** CLL cells were treated with calcitriol and viability was assessed by flow cytometry at 24 h of cell culture. No differences were identified between control and calcitriol-treated cultures. Bars represent the median values and the Wilcoxon test was performed to assess statistical significance. ns: not significant.

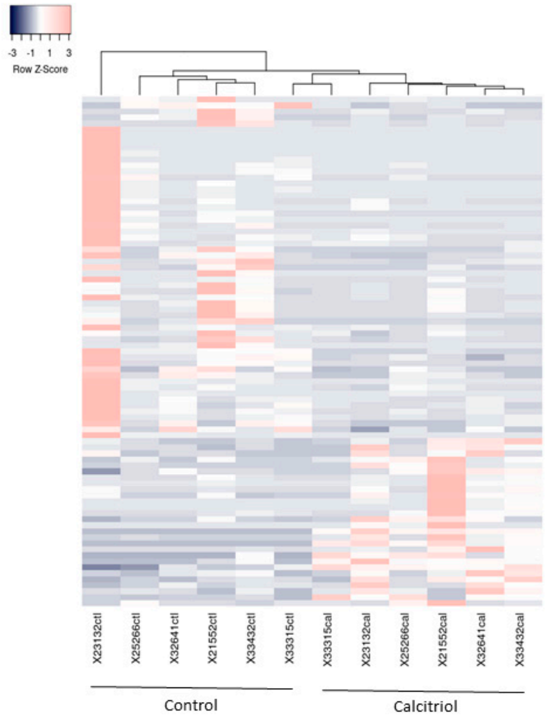

Figure S3. Transcriptome analysis. Supervised hierarchical clustering based on significantly differentially expressed genes.

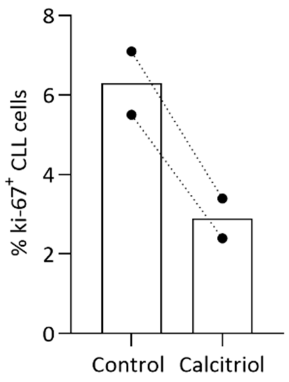

Figure S4. Proliferation assay. Flow cytometric analysis of proliferating cells (Ki-67+) in two U-CLL cases co-cultured with HS5 stromal cells for 4 days and supplemented with calcitriol at 48 h of culture.

Table S1. Survival analysis for the correlation of VDR mRNA expression with Overall Survival (OS) and Time-To-First Treatment (TTFT) in CLL cases.

	VDR High/Low CLL Patients	Number of CLL Patients	Median (Months)	Logrank
OS	VDR^{high} patients > 0.000316	47	98	0.0415
	VDR^{low} patients < 0.000316	23	118	
TTFT	VDR^{high} patients > 0.000557	36	39	0.0019
	VDR^{low} patients < 0.000557	34	83.5	

Table 2. List of all 85 significantly up- and down-regulated genes in CLL cells following treatment with calcitriol.

**Up-Regulated Genes**			**Down-Regulated Genes**					
**Gene Name**	**log2FC**	***p* Value**	**Gene Name**	**Log2FC**	***p* Value**	**Gene Name**	**log2FC**	***p* Value**
CYP24A1	9.75	6.12×10^{-8}	ACTR3BP2	−6.24	0.038015738	FABP4	−3.82	0.023904066
PVALB	4.59	0.008923563	ELAVL2	−6.24	0.038015738	RND3	−3.51	0.046345681
SPOCK1	4.52	0.003704385	OVCH1-AS1	−6.07	0.04234957	PPP2R3B	−3.40	0.000575751
AQP9	4.45	0.027368354	TMIGD3	−6.05	0.002883498	CHST13	−3.33	0.012028134
CAMP	4.26	1.57×10^{-5}	PGM5P3-AS1	−5.94	0.048643137	ADAMTSL5	−3.29	0.018961077
IGFN1	4.17	0.02075608	LOC284788	−5.94	0.048643137	VAMP7	−3.25	0.00270883
LAMC2	4.16	0.003187541	CADPS2	−5.25	0.04583426	SNORA47	−3.25	0.027629819
LOC101928222	3.62	0.014259383	C5orf64	−5.23	0.046596979	SCIN	−3.22	0.042277042
LINC01554	3.41	0.02098677	PARVA	−5.04	0.01775823	ZFX-AS1	−3.10	0.019457071
HTRA1	3.10	0.021521139	COL14A1	−5.01	0.035833445	ADORA3	−2.98	0.008062504
C20orf197	3.02	0.038036937	RNU6-79P	−4.96	0.025746732	GCSAML	−2.83	0.007154774
RASIP1	2.83	0.038423558	LOC442497	−4.82	0.013861637	CDKL5	−2.79	0.014003301

CD14	2.79	0.037391158	STAB1	−4.78	0.000996363	S100A9	−2.67	0.040961187
CD248	2.78	0.04994425	TSKS	−4.77	0.019350897	TULP2	−2.66	0.04379134
FAIM2	2.59	0.002924506	C1QC	−4.76	0.002160563	DEPDC1B	−2.42	0.028224961
RGL2	2.54	9.44×10^{-5}	EDNRB	−4.65	0.010729171	GPR21	−2.38	0.048568413
GAGE10	2.44	0.045516179	SENP3-EIF4A1	−4.60	0.030013135	CUTA	−2.35	0.004702445
STEAP3	2.09	0.012623635	UNC13A	−4.57	0.033373306	ASB9P1	−2.27	0.03636743
DPP4	1.96	0.041621599	DCDC5	−4.48	0.03030353	TMEM184A	−2.24	0.013132614
LGALS9B	1.89	0.028021494	CELF5	−4.46	0.010955212	S100A8	−2.09	0.020777944
FN1	1.81	0.000867989	LILRA5	−4.42	0.037897313	SYT1	−1.94	0.023162401
EPB41L1	1.70	0.016969034	MIR4753	−4.03	0.007714012	LOC101927768	−1.91	0.048931744
NRADDP	1.70	0.037811997	RAB3IL1	−4.02	0.03913515	LGMN	−1.90	0.047992609
YTHDC1	1.38	0.014443782	KITLG	−4.00	0.004411518	ZNF157	−1.59	0.049976482
HTT-AS	1.38	0.023242301	HS3ST2	−3.94	0.032667428	LOC101927211	−1.31	0.034780453

DENND6B	1.31	0.006684191	G6PC	−3.92	0.013243736	CORIN	−1.27	0.017722845
PLXNB2	1.23	0.016271863	VWA8-AS1	−3.91	0.045515336	SMA5	−1.24	0.048538344
GEM	1.16	0.020430631	TINAGL1	−3.89	0.022432124	CAMK1	−1.24	0.026136777
						LRRC70	−1.18	0.027992735

Table S3. Clinicobiological data for the CLL patients' study group.

Patient ID	Age at Diagnosis	Sex	Rai at Diagnosis	Binet at Diagnosis	Surface IGH Isotype	Mutational Status of IGHV Genes	Ibrutinib Treated
P10029	57	F	I	A	MD	Unmutated	No
P10193	81	M	0	A	MD	Unmutated	No
P103	45	F	0	A	G	Mutated	No
P10393	55	M	0	A	MD	Mutated	No
P11323	67	M	0	A	MD	Unmutated	No
P11657	47	M	II	A	MD	Unmutated	No
P11722	76	M	II	A	MD	Unmutated	No
P1188	58	M	II	B	MD	Mutated	No
P12206	65	M	I	A	MD	Unmutated	No
P12321	42	M	0	A	G	Mutated	No
P14197	49	M	II	B	MD	Unmutated	No
P1615	62	F	0	A	G	Unmutated	No
P1626	37	M	0	A	G	Mutated	No
P18198	74	M	0	A	MD	Unmutated	No
P18384	62	M	0	A	MD	Mutated	No
P1894	53	M	0	A	M	Mutated	No
P23503	43	M	0	A	MD	Unmutated	No
P2355	54	M	II	A	MD	Unmutated	No
P23587	67	F	0	A	MD	Unmutated	No
P23927	66	M	I	A	M	Unmutated	No
P2528	52	F	I	A	MD	Mutated	No
P2920	51	M	0	A	G	Mutated	No
P3020	66	F	0	A	G	Mutated	No
P32166	75	M	0	A	MD	Mutated	No
P3506	71	M	0	A	MD	Unmutated	No
P3551	58	F	0	A	G	Mutated	No
P3870	68	M	0	A	MD	Unmutated	No
P3966	70	F	I	A	MD	Unmutated	No
P4557	58	F	0	A	MD	Mutated	No

P4700	41	F	II	B	M	Unmutated	No
P511	64	M	II	B	MD	Unmutated	No
P5610	47	F	0	A	MD	Mutated	No
P5728	53	M	I	A	MD	Mutated	No
P573	68	M	0	A	MD	Unmutated	No
P585	48	M	0	A	MD	Mutated	No
P5895	67	M	0	A	MD	Mutated	No
P6077	74	M	IV	C	MD	Unmutated	No
P6124	68	M	0	A	MD	Unmutated	No
P6460	70	M	0	A	MD	Mutated	No
P7155	51	M	0	A	MD	Unmutated	No
P7317	80	M	III	C	MD	Unmutated	No
P7395	73	M	I	A	MD	Unmutated	No
P7648	75	F	0	A	MD	Unmutated	No
P7981	66	M	I	A	MD	Unmutated	No
P8458	53	M	I	A	G	Mutated	No
P8762	63	M	I	A	MD	Unmutated	No
P8979	50	M	0	A	MD	Mutated	No
P9001	67	M	I	A	MD	Unmutated	No
P9391	83	M	III	C	MD	Mutated	No
P9881	60	F	0	A	G	Mutated	No
P9208	43	M	I	A	G	Mutated	No
P608	53	M	0	A	MD	Mutated	No
P571	48	M	0	A	MD	Unmutated	No
P1173	62	F	I	A	MD	Unmutated	Yes
P3041	64	M	II	A	MD	Unmutated	No
P8655	66	M	II	B	MD	Unmutated	No
P1618	62	M	0	A	MD	Mutated	No
P1939	44	M	II	B	G	Mutated	No
P20460	79	M	0	A	MD	Unmutated	No
P3073	38	F	IV	C	MD	Unmutated	No
P3560	57	F	I	A	MD	Unmutated	No
P4383	70	F	0	A	G	Mutated	No

P5017	71	F	0	A	MD	Unmutated	No
P5359	58	F	0	A	MD	Mutated	No
P5588	61	F	0	A	MD	Unmutated	No
P6520	45	M	0	A	G	Mutated	No
P711	65	M	II	B	MD	Mutated	Yes
P7929	38	F	I	A	MD	Mutated	No
P7937	66	M	I	A	MD	Unmutated	No
P13792	68	M	0	A	MD	Mutated	No
P21610	47	M	I	A	N/A	Unmutated	Yes
P12336	60	M	II	A	MD	Unmutated	Yes
P30572	N/A	M	N/A	N/A	N/A	Unmutated	Yes
P18780	61	F	0	A	M	Unmutated	Yes
P9320	47	M	I	A	MD	Unmutated	Yes
P21499	61	M	I	A		Unmutated	Yes
P8800	57	M	0	A	MD	Unmutated	Yes
P22054	73	F	0	A	MD	Unmutated	Yes
P8755	69	M	II	B	M	Unmutated	Yes
P3492	39	M	I	A	G	Unmutated	Yes
P23132	68	M	I	A	MD	Unmutated	No
P18625	63	F	0	A	MD	Unmutated	No
P32088	63	M	II	B	MD	Unmutated	No
P12580	90	F	I	A	MD	Unmutated	No
P15217	66	M	I	B	MD	Unmutated	No
P21552	71	M	0	A	MD	Unmutated	No
P25835	69	M	I	A	MD	Unmutated	No
P20443	49	F	I	A	N/A	Unmutated	No
P11159	62	M	0	A	MD	Mutated	No
P22229	36	F	0	A	MD	Mutated	No
P6638	66	F	I	A	MD	Mutated	No
P2685	68	F	0	A	N/A	Mutated	No
P5949	50	M	0	A	MD	Mutated	No

P16705	78	F	0	A	MD	Unmutated	No

Table S4. List of antibodies used for flow cytometry analysis.

Antibody	Conjugate	Company
VDR (D-6)	PE	Santa Cruz Biotechnology
CYP24 (E-7)	FITC	Santa Cruz Biotechnology
ERK1/2 (pT202/pY204)	Alexe Fluor 488	BD Biosciences
NF-κB p65 (pS529)	PE	BD Biosciences
CD19	PE-Cy5	BD Biosciences
IgG2α	PE	Beckman Coulter
IgG1	FITC	BD Biosciences
IgG2b, κ	PE	BioLegend

Publisher's Note: MDPI stays neutral with regard to jurisdictional claims in published maps and institutional affiliations.

© 2020 by the authors. Licensee MDPI, Basel, Switzerland. This article is an open access article distributed under the terms and conditions of the Creative Commons Attribution (CC BY) license (http://creativecommons.org/licenses/by/4.0/).
